# Efficacy and diabetes risk of moderate-intensity statin plus ezetimibe versus high-intensity statin after percutaneous coronary intervention

**DOI:** 10.1186/s12933-024-02498-3

**Published:** 2024-11-05

**Authors:** Eun Ho Choo, Donggyu Moon, Ik Jun Choi, Sungmin Lim, Jungkuk Lee, Dongwoo Kang, Byung-Hee Hwang, Chan Joon Kim, Jong-Min Lee, Ki-Dong Yoo, Doo Soo Jeon, Kiyuk Chang

**Affiliations:** 1 Division of Cardiology, Department of Internal Medicine, Seoul St. Mary’s Hospital, College of Medicine, The Catholic University of Korea, Seoul, Republic of Korea; 2 Catholic Research Institute for Intractable Cardiovascular Disease (CRID), College of Medicine, The Catholic University of Korea, Seoul, Republic of Korea; 3 Division of Cardiology, Department of Internal Medicine, St Vincent’s Hospital, College of Medicine, The Catholic University of Korea, Seoul, Republic of Korea; 4 Division of Cardiology, Department of Internal Medicine, Incheon St. Mary’s Hospital, College of Medicine, The Catholic University of Korea, 56 Dongsu-ro, Bupyeong-dong, Bupyeong-gu, 21431 Incheon, Seoul, Republic of Korea; 5 Division of Cardiology, Department of Internal Medicine, Uijeongbu St. Mary’s Hospital, College of Medicine, The Catholic University of Korea, 271, Cheonbo-ro, 11765 Uijeongbu-si, Seoul, Gyeonggi-do Republic of Korea; 6 Data Science Team, Hanmi Pharm. Co., Ltd, Seoul, Republic of Korea

**Keywords:** Coronary artery disease, Dyslipidemia, Ezetimibe, Statin, Percutaneous coronary intervention

## Abstract

**Backgrounds:**

High-intensity statin is recommended for patients undergoing percutaneous coronary intervention (PCI), and ezetimibe is recommended to be added in patients not achieving low-density lipoprotein cholesterol (LDL-C) targets. Moderate-intensity statin plus ezetimibe can reduce LDL-C levels similar to high-intensity statin. The aim of this study is to examine the long-term efficacy and safety of moderate-intensity statin plus ezetimibe as the first-line strategy compared to high-intensity statin in patients undergoing PCI.

**Method:**

Data was obtained from the Health Insurance Review and Assessment Service database of South Korea. Patients who underwent PCI from 2012 to 2017 were included. The primary efficacy endpoint was major adverse cardiac cerebrovascular events (MACCEs), a composite of all-cause death, revascularization, or ischemic stroke. The safety endpoint was new-onset diabetes mellitus (DM).

**Results:**

A total of 45,501 patients received high-intensity statin (*n* = 38,340) or moderate-intensity statin plus ezetimibe (*n* = 7,161). Among propensity-score-matched 7,161 pairs, MACCEs occurred in 1,460 patients with high-intensity statin and 1,406 patients with moderate-intensity statin plus ezetimibe (33.8% vs. 31.9%, hazard ratio 0.96, 95% confidence interval 0.89–1.03, *P* = 0.27) at a median follow-up of 2.7 years. DM was newly diagnosed in 398 patients with high-intensity statin and 342 patients with moderate-intensity statin plus ezetimibe (12.5% vs. 10.7%; hazard ratio 0.84, 95% confidence interval 0.73–0.97, *P* = 0.02).

**Conclusion:**

In patients undergoing PCI, moderate-intensity statin plus ezetimibe demonstrated a similar risk of MACCEs but a lower risk of new-onset DM than high-intensity statin. Early combination treatment of moderate-intensity statin and ezetimibe may be a useful and safe lipid-lowering strategy after PCI.

**Graphical abstract:**

Cumulative incidence according to the first-line lipid-lowering strategy in patients undergoing percutaneous coronary intervention. Abbreviation: MACCE, major adverse cardiac cerebrovascular events; PCI, percutaneous coronary intervention.
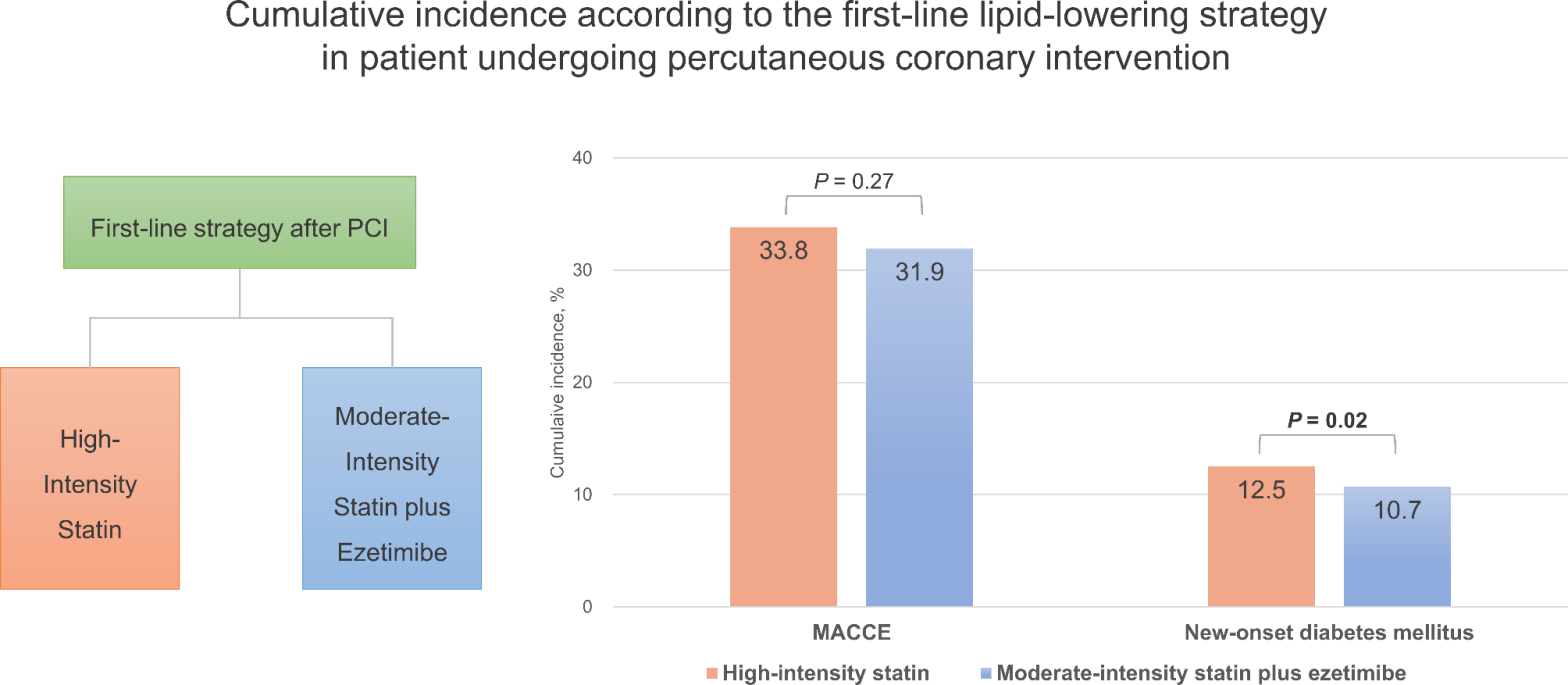

**Supplementary Information:**

The online version contains supplementary material available at 10.1186/s12933-024-02498-3.

## Background

Current guidelines emphasize the importance of achieving a low-density lipoprotein-cholesterol (LDL-C) target of less than 55 mg/dL and a reduction of more than 50% from baseline for patients undergoing percutaneous coronary intervention (PCI) [1]. However, achieving these stringent targets remains challenging with high-intensity statin therapy alone, which is the recommended first-line treatment.

Studies have demonstrated that many patients who undergo PCI do not reach the target LDL-C levels [2]. Increasing the statin dose to address this issue is associated with adverse effects such as statin-associated myalgia, liver enzyme elevation, and, notably, a dose-dependent increase in the risk of new-onset diabetes over the long term [3, 4]. Moreover, doubling the statin dose yields only an additional 6–7% reduction in LDL-C [5]. 

Ezetimibe, a cholesterol absorption inhibitor, can reduce LDL-C by an additional 15–22% when added to statin [1, 6]. The combination of ezetimibe with moderate-intensity statins has been shown to achieve greater LDL-C reduction than doubling the dose of moderate-intensity statins to high-intensity statins [7, 8]. Additionally, this combination improves glucose metabolism by reducing insulin resistance and hemoglobin A1c, while effectively lowering LDL-C [9]. 

Given the potential side effects of high-intensity statin, starting with a combination of ezetimibe and moderate-intensity statin from the index PCI may offer a more effective and safer treatment strategy. This study aims to explore the long-term efficacy and safety of this combination therapy as the first-line strategy for patients undergoing PCI in real-world practice.

## Methods

### Study design and data source

This study is a population-based retrospective nationwide cohort study conducted by using claim data from the Health Insurance Review and Assessment (HIRA) Service. South Korea has a single-payer national health system. Most Korean people are required to be enrolled in the National Health Insurance Service. The HIRA is a government organization that examines the claim data, appropriateness of medical costs, and health care service quality. All healthcare providers in South Korea are mandated to participate in this system and use the resident identification numbers and unique codes to claim medical costs to receive reimbursement. The HIRA data includes demographic information, diagnosis codes, surgical and procedural codes, therapeutical devices, and prescription drug details such as name, dose, usage, and date. The diagnoses are coded in accordance with the Korean Standard Classification of Diseases Version 6, which is based on the International Classification of Diseases 10th Revision (ICD-10). The HIRA provides anonymized and customized research data after reviews by a committee. The study protocol was approved by the Institutional Review Board at the Uijeongbu St. Mary’s Hospital, The Catholic University of Korea (IRB Number: UC20ZISE0031). Informed consent was not considered necessary because the patient records and information were anonymized and de-identified before analysis.

### Study population

This study included patients over 20 years old who underwent PCI (M6551‒6554, M6561‒6567, M6571, M6572, and M6638) with indication of acute myocardial infarction (I21 or I22) or angina (I20, I24, or I25) between January 2012 and December 2017 and who were prescribed statins after the index PCI. To compare the long-term effect of high-intensity statin and moderate-intensity statin plus ezetimibe as a first-line strategy following PCI, we excluded patients with the following criteria: (1) those who died in hospital, (2) those who were prescribed other lipid-lowering agents, (3) those who were prescribed statin less than 80% of days (24 days) in the first month after the index PCI, (4) those who were prescribed only moderate- or low-intensity statin or other combinations of statins and ezetimibe, (5) those whose statin intensity or combination had changed to a different type within 1 year, and (6) those who were lost to follow-up within 1 year, as the claim data was not available for study assignment and analyses. The intensity of statins was defined according to current guidelines and divided into the following categories [1]. High-intensity statin included atorvastatin 40 mg or 80 mg and rosuvastatin 20 mg or 40 mg, while moderate-intensity statin included simvastatin 20 mg or 40 mg, lovastatin 40 mg, pravastatin 40 or 80 mg, fluvastatin 80 mg, atorvastatin 10 mg or 20 mg, rosuvastatin 5 mg or 10 mg, and pitavastatin 1, 2, or 4 mg. The ezetimibe combination therapy includes a single combination pill and separate pills.

### Outcomes and definitions

The primary efficacy endpoint was major adverse cardiac cerebrovascular events (MACCEs), a composite of all-cause death, revascularization, or ischemic stroke. We also evaluated the individual components of MACCE. Death was defined as either a medical treatment termination code indicating in-hospital death or the absence of any medical claims for more than one year, presuming death with over 95% accuracy [10]. Revascularization was divided into three categories: PCI due to myocardial infarction, PCI due to angina, and coronary artery bypass graft. These events were determined based on inpatient ICD-10 codes and procedural codes 30 days after the index PCI. Any revascularization within 30 days was considered the index PCI-related procedure. Detailed definitions of comorbidities and clinical outcomes were presented in Supplementary Table 1.

The safety endpoints were new-onset diabetes mellitus (DM) with medications, intracranial hemorrhage, use of hepatoprotective agents (drugs used to mitigate or prevent various forms of liver injury), and rhabdomyolysis. New-onset DM was analyzed after excluding patients with a history of DM with medications and patients who have received new anti-diabetic medications or insulin within 30 days after the index PCI. The specific names and codes of each medication used in the analyses are provided in Supplementary Table 2. Korean National Health Insurance system, anti-diabetic medications are covered only for patients diagnosed with diabetes. SGLT2 inhibitors have been covered for heart failure since 2023, and GLP-1 receptor agonists have not been covered for weight loss. Therefore, the likelihood of these medications being prescribed for non-diabetes reasons in our study period is minimal.

The Charlson Comorbidity Index was employed to summarize comorbidities based on the ICD-10 diagnosis codes. Given the nature of the claim data used in this study, adherence was determined based on the filling of scripts, as claim data are only recorded when prescriptions are filled. Good adherence was defined as filling prescriptions for 80% or more days of high-intensity statin or moderate-intensity statin plus ezetimibe in the first year after the index PCI, while poor adherence was defined as filling prescriptions for less than 80% of days.

### Statistical analysis

Continuous variables were reported as mean ± standard deviation or median (interquartile range) and analyzed using independent samples Student’s *t*-test or the Mann-Whitney *U* test. Categorical variables were expressed as percentages or rates and analyzed using the chi-square or Fisher exact test. To minimize the impact of bias and the potential confounding factor for comparing two first-line lipid-lowering strategies, a propensity score analysis was performed. A propensity score was calculated by multiple logistic regression models, and all baseline characteristics in Table 1 were included as variables in the propensity score estimation. According to the propensity score, patients were selected by 1:1 matching without replacement using the nearest neighbor method, and a caliper width of 0.2 standardized differences was used for matching. Kaplan-Meier analysis was used to evaluate the cumulative incidence of clinical events, and a comparison of outcomes between groups was performed using the log-rank test. Cox proportional hazard models were applied to estimate hazard ratio (HR) and 95% confidence interval (CI) for endpoints. Subgroup analyses were conducted in the propensity score-matched population and based on age, sex, history of diabetes mellitus with medications, previous statin use, history of previous PCI, stroke, or chronic kidney disease, degrees of hospital, clinical diagnosis at the index PCI, and number of implanted stents more than one. In the multivariable Cox regression model, the following covariates were adjusted: statin, age, sex, and the variables that were significantly different at univariate analyses (*P* < 0.10). In addition, these variables were also adjusted to reduce the bias from different degrees of hospitals, the year of the index PCI, and the compliance with the statin. As an additional sensitivity analysis, clinical outcomes according to treatment were investigated in patients with good adherence. After excluding patients with poor adherence, a new propensity score was calculated, and a new 1:1 matching was performed. All analyses were 2-tailed, and P values < 0.05 were considered to indicate statistical significance. All statistical analyses were performed using g SAS software version 9.4 (SAS Institute Inc., Cary, NC, USA) and R version 3.6.1 (R Foundation for Statistical Computing).

**Table 1:** Baseline characteristics in study population

Variables	Crude population				Propensity score-matched population			
	High-intensity statin (*n* = 38,340)	Moderate-intensity statin plus ezetimibe (*n* = 7,161)	*P* value		High-intensity statin (*n* = 7,161)	Moderate-intensity statin plus ezetimibe (*n* = 7,161)	*P* value	Standardized difference
Age	61.88 ± 11.48	64.11 ± 10.59	< 0.001		64.17 ± 10.53	64.11 ± 10.59	0.74	-0.006
Age			< 0.001				0.98	
<55	10,425 (27.2)	1349 (18.8)			1331 (18.6)	1349 (18.8)		
55–64	12,021 (31.4)	2250 (31.4)			2269 (31.7)	2250 (31.4)		
65–74	10,030 (26.2)	2317 (32.4)			2316 (32.3)	2317 (32.4)		
75≤	5864 (15.3)	1245 (17.4)			1245 (17.4)	1245 (17.4)		
Male	29,493 (76.9)	5131 (71.7)	< 0.001		5180 (72.3)	5131 (71.7)	0.36	-0.015
Hypertension with medication	24,879 (64.9)	5461 (76.3)	< 0.001		5399 (75.4)	5461 (76.3)	0.23	0.020
Diabetes mellitus with medication	10,475 (27.3)	2374 (33.2)	< 0.001		2396 (33.5)	2374 (33.2)	0.70	-0.007
Previous statin	20,951 (54.6)	5102 (71.2)	< 0.001		5031 (70.3)	5102 (71.2)	0.19	0.022
Previous myocardial infarction	2234 (5.8)	602 (8.4)	< 0.001		611 (8.5)	602 (8.4)	0.79	-0.005
Previous percutaneous coronary intervention	2007 (5.2)	695 (9.7)	< 0.001		697 (9.7)	695 (9.7)	0.96	-0.001
Previous coronary artery bypass graft	26 (0.1)	10 (0.1)	0.05		8 (0.1)	10 (0.1)	0.64	0.008
Previous ischemic stroke	3385 (8.8)	743 (10.4)	< 0.001		733 (10.2)	743 (10.4)	0.78	0.005
Previous intracranial hemorrhage	266 (0.7)	68 (0.9)	0.02		67 (0.9)	68 (0.9)	0.93	0.001
Heart failure	4273 (11.1)	1004 (14)	< 0.001		989 (13.8)	1004 (14)	0.72	0.006
Atrial fibrillation	1046 (2.7)	317 (4.4)	< 0.001		297 (4.1)	317 (4.4)	0.41	0.014
Peripheral artery disease	1741 (4.5)	455 (6.4)	< 0.001		466 (6.5)	455 (6.4)	0.71	-0.006
Chronic kidney disease	4419 (11.5)	1120 (15.6)	< 0.001		1099 (15.3)	1120 (15.6)	0.63	0.008
Chronic obstructive pulmonary disease	1104 (2.9)	199 (2.8)	0.64		195 (2.7)	199 (2.8)	0.84	0.003
Malignancy	1327 (3.5)	269 (3.8)	0.21		263 (3.7)	269 (3.8)	0.79	0.004
Chronic liver disease	4132 (10.8)	944 (13.2)	< 0.001		929 (13)	944 (13.2)	0.71	0.006
Peptic ulcer disease	3905 (10.2)	964 (13.5)	< 0.001		950 (13.3)	964 (13.5)	0.73	0.006
Dementia	1505 (3.9)	420 (5.9)	< 0.001		416 (5.8)	420 (5.9)	0.89	0.002
Connective tissue disease	686 (1.8)	144 (2.0)	0.20		137 (1.9)	144 (2.0)	0.67	0.007
Rhabdomyolysis	54 (0.1)	14 (0.2)	0.27		12 (0.2)	14 (0.2)	0.69	0.007
Charlson Comorbidity Index	2.01 ± 1.76	2.43 ± 1.89	< 0.001		2.41 ± 1.86	2.43 ± 1.89	0.72	0.006
Charlson Comorbidity Index			< 0.001				0.55	
0	7887 (20.6)	1012 (14.1)			1026 (14.3)	1012 (14.1)		
1	9666 (25.2)	1603 (22.4)			1532 (21.4)	1603 (22.4)		
2	8263 (21.6)	1556 (21.7)			1568 (21.9)	1556 (21.7)		
3≤	12,524 (32.7)	2990 (41.8)			3035 (42.4)	2990 (41.8)		
Clinical diagnosis at the index procedure			< 0.001				0.95	-0.001
Acute myocardial infarction	19,162 (50)	1641 (22.9)			1644 (23)	1641 (22.9)		
Angina pectoris	19,178 (50)	5520 (77.1)			5517 (77)	5520 (77.1)		
Number of implanted stents	1.43 ± 0.87	1.38 ± 0.91	< 0.001		1.39 ± 0.87	1.38 ± 0.91	0.64	-0.008
Number of implanted stents			0.05				0.59	
1	25,548 (66.6)	4857 (67.8)			4827 (67.4)	4857 (67.8)		
2≤	12,792 (33.4)	2304 (32.2)			2334 (32.6)	2304 (32.2)		
Hospital			0.47				0.87	-0.003
Tertiary center	18,918 (49.3)	3567 (49.8)			3577 (50)	3567 (49.8)		
Primary or secondary center	19,422 (50.7)	3594 (50.2)			3584 (50)	3594 (50.2)		
Year of the index percutaneous coronary intervention			< 0.001				0.50	
2012	3011 (7.9)	1093 (15.3)			1139 (15.9)	1093 (15.3)		
2013	3890 (10.1)	943 (13.2)			959 (13.4)	943 (13.2)		
2014	6557 (17.1)	722 (10.1)			731 (10.2)	722 (10.1)		
2015	8168 (21.3)	764 (10.7)			742 (10.4)	764 (10.7)		
2016	9025 (23.5)	1460 (20.4)			1374 (19.2)	1460 (20.4)		
2017	7689 (20.1)	2179 (30.4)			2216 (30.9)	2179 (30.4)		
Medication								
Aspirin	37,858 (98.7)	6857 (95.8)	< 0.001		6896 (96.3)	6857 (95.8)	0.10	-0.028
Clopidogrel	24,799 (64.7)	5630 (78.6)	< 0.001		5638 (78.7)	5630 (78.6)	0.87	0.023
Prasugrel	2307 (6.0)	448 (6.3)	0.44		470 (6.6)	448 (6.3)	0.45	0.007
Ticagrelor	10,752 (28.0)	779 (10.9)	< 0.001		788 (11.0)	779 (10.9)	0.81	-0.050
Beta-blocker	29,718 (77.5)	4379 (61.2)	< 0.001		4387 (61.3)	4379 (61.2)	0.89	-0.002
Renin-angiotensin system inhibitor	26,162 (68.2)	3333 (46.5)	< 0.001		3339 (46.6)	3333 (46.5)	0.92	-0.002
Warfarin	915 (2.4)	128 (1.8)	0.002		134 (1.9)	128 (1.8)	0.71	-0.006
Direct oral anticoagulants	612 (1.6)	116 (1.6)	0.88		115 (1.6)	116 (1.6)	0.95	0.001

Data are presented as mean ± standard deviation or number (percentage)

## Results

### Study population and characteristics

**Fig. 1:**
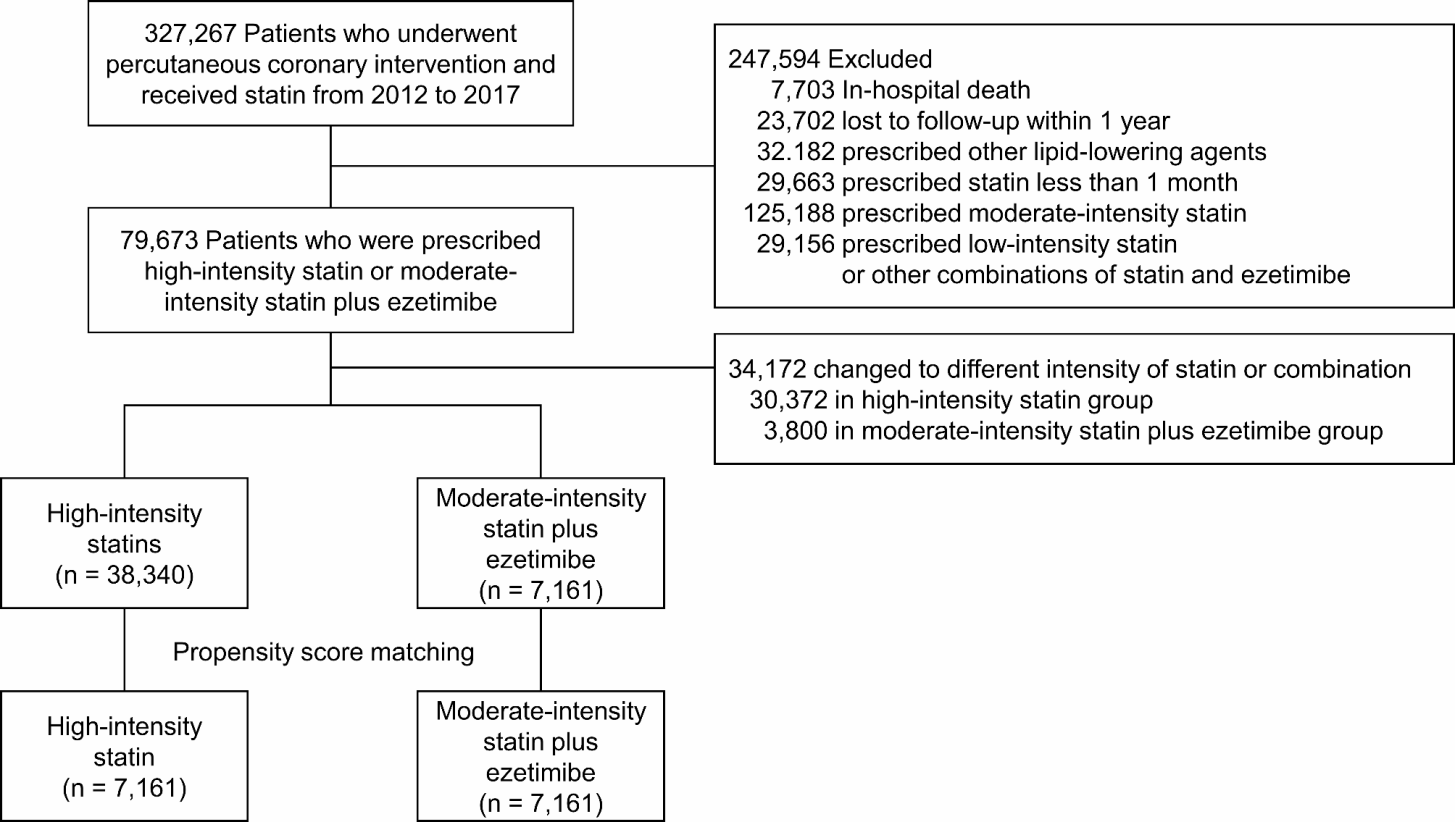
Study flow chart

Between 2012 and 2017, a total of 327,267 patients undergoing PCI were identified from the HIRA claims database. Among them, 45,501 patients met the eligibility criteria and were included in the study (Fig. 1). The mean age of the study population was 62.2 years, with 34,624 (76.1%) being male. In the crude population, the moderate-intensity statin plus ezetimibe group was older, had a higher Charlson Comorbidity Index score, and a smaller proportion of patients with acute myocardial infarction compared to the high-intensity statin group. After 1:1 propensity score matching, the data from a total of 14,322 patients was analyzed. Table 1 provides the baseline characteristics of both the overall study population and the propensity-score matched population (Table 1).

### Clinical outcomes

**Fig. 2:**
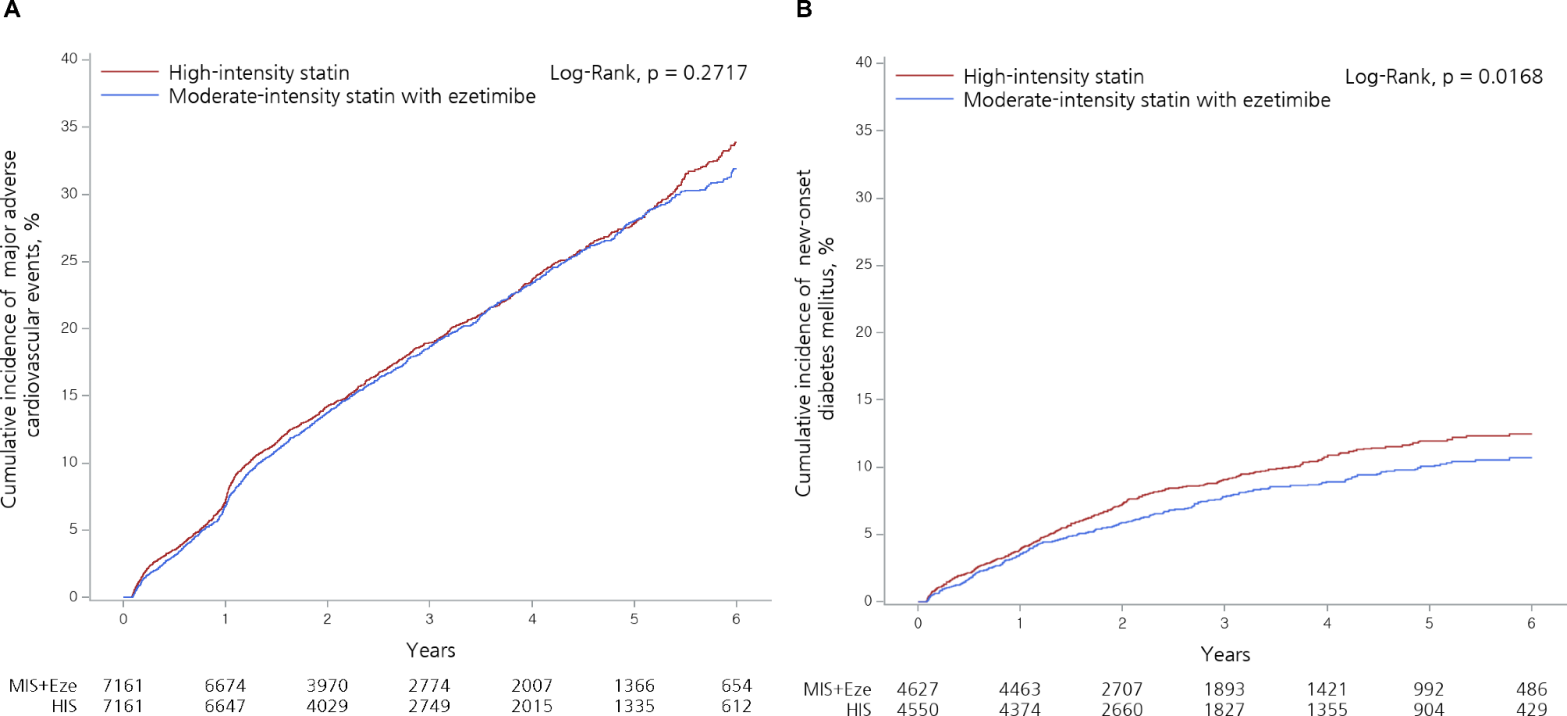
Six-year Kaplan-Meier estimated percentage for (A) major adverse cardiac cerebrovascular events and (B) new-onset diabetes mellitus in the propensity score-matched population. Abbreviation: HIS, high-intensity statin; MIS + Eze, moderate-intensity statin plus ezetimibe

Clinical outcomes according to treatment group are listed in Table 2 (propensity-score matched population) and Supplementary Table 3 (crude population). In the propensity-score matched population, with a median follow-up period of 2.7 years (interquartile range 1.6–4.9), the incidence of the primary efficacy endpoint (MACCE) was similar between the two groups (HR, 0.96; 95% CI, 0.89–1.03, *P* = 0.27). The incidences of the individual components of the primary efficacy endpoint were also similar between the groups. For safety endpoints, the incidence of new-onset DM was significantly lower in the moderate-intensity statin plus ezetimibe group than high-intensity statin group (HR, 0.84; 95% CI, 0.73–0.97; *P* = 0.02). The incidences of other safety endpoints, such as intracranial hemorrhage, use of hepatoprotective agents, and rhabdomyolysis, were similar between the groups. Figure 2 depicts the cumulative incidence of long-term MACCEs and new-onset DM between the two groups.

**Table 2:** Clinical outcomes in the propensity score-matched population

Endpoints	Cumulative incidence^*^		Incidence rate per 100 person-year		Log-rank *P* value	HR (95% CI)	*P* value
	High-intensity statin (*N* = 7,161)	Moderate-intensity statin plus ezetimibe (*N* = 7,161)	High-intensity statin (*N* = 7,161)	Moderate-intensity statin plus ezetimibe (*N* = 7,161)			
Efficacy endpoints							
Major adverse cardiac cerebrovascular events^†^	1,460 (33.8)	1.406 (31.9)	7.1	6.8	0.27	0.96 (0.89‒1.03)	0.27
All-cause death	456 (14.8)	448 (13.9)	2.0	2.0	0.92	0.99 (0.87‒1.13)	0.92
Revascularization	831 (18.0)	781 (16.8)	3.9	3.7	0.19	0.94 (0.85‒1.03)	0.19
Percutaneous coronary intervention due to myocardial infarction	128 (3.2)	115 (2.9)	0.6	0.5	0.42	0.90 (0.70‒1.16)	0.42
Percutaneous coronary intervention due to angina	697 (14.6)	659 (13.8)	3.2	3.1	0.30	0.95 (0.85‒1.05)	0.30
Coronary artery bypass graft	13 (0.4)	12 (0.4)	0.1	0.1	0.87	0.94 (0.43‒2.05)	0.87
Ischemic stroke	381 (8.8)	396 (8.9)	1.7	1.8	0.53	1.05 (0.91‒1.20)	0.53
Transient ischemic attack	70 (1.6)	80 (2.0)	0.3	0.4	0.39	1.15 (0.84‒1.59)	0.39
Cerebral infarction	332 (7.7)	339 (7.5)	1.5	1.5	0.73	1.03 (0.88‒1.20)	0.73
Safety endpoints							
New-onset diabetes mellitus with medications	398 (12.5)	342 (10.7)	2.9	2.5	0.02	0.84 (0.73‒0.97)	0.02
Intracranial hemorrhage	84 (2.0)	97 (2.3)	0.4	0.4	0.30	1.17 (0.87‒1.56)	0.30
Hepatoprotective agents	366 (9.5)	396 (10.2)	1.6	1.8	0.20	1.10 (0.95‒1.27)	0.20
Rhabdomyolysis	29 (0.7)	27 (0.8)	0.1	0.1	0.81	0.94 (0.56‒1.58)	0.81

^*^Events reported as number (6-year Kaplan-Meier estimate)

^†^Composite of all-cause death, revascularization, or stroke

### Predictors of ischemic events and new-onset diabetes

Table 3 demonstrates factors associated with the risk of the occurrence of MACCE or new-onset DM in the Cox regression model. Factors such as age, hypertension, and chronic obstructive pulmonary disease were significant predictors for both MACCE and new-onset DM. Notably, there was a relationship between drug adherence and risk for clinical events. Compared with patients with poor adherence, those with good adherence had a significantly lower risk of MACCE (adjusted HR 0.56; 95% CI, 0.52–0.59). Good adherence to statin was inversely correlated with new-onset DM (adjusted HR, 1.49; 95% CI, 1.25–1.77). The risk of new-onset DM was higher in patients who were already on statin treatment, whereas the risk was lower in patients who had recently undergone PCI, indicating a shorter duration of statin treatment.

**Table 3:** Unadjusted and adjusted hazard ratios for the clinical events in crude population

	Major adverse cardiac cerebrovascular events^*^				New-onset diabetes mellitus			
	Unadjusted HR (95% CI)	P value	Adjusted HR (95% CI)	P value	Unadjusted HR (95% CI)	P value	Adjusted HR (95% CI)	P value
Moderate intensity statin plus ezetimibe (vs. high-intensity statin)	0.99 (0.93‒1.04)	0.63	0.98 (0.92‒1.04)	0.51	0.83 (0.74‒0.93)	0.001	0.82 (0.73‒0.92)	0.001
Age	1.02 (1.02‒1.02)	< 0.001	1.01 (1.01‒1.01)	< 0.001	0.99 (0.99‒1.00)	< 0.001	0.99 (0.99‒1.00)	< 0.001
Female (vs. male)	1.03 (0.98‒1.08)	0.29	0.87 (0.83‒0.92)	< 0.001	0.97 (0.88‒1.06)	0.45		
Hypertension	1.33 (1.27‒1.40)	< 0.001	1.07 (1.02‒1.13)	0.009	1.14 (1.05‒1.23)	0.001	1.16 (1.06‒1.27)	0.001
Diabetes mellitus	1.33 (1.27‒1.39)	< 0.001	1.13 (1.08‒1.19)	< 0.001	‒	‒	‒	‒
Previous statin	1.12 (1.08‒1.17)	< 0.001			1.11 (1.03‒1.20)	0.006	1.11 (1.02‒1.21)	0.02
Previous myocardial infarction	1.23 (1.14‒1.33)	< 0.001			1.02 (0.87‒1.20)	0.81		
Previous percutaneous coronary intervention	1.35 (1.26‒1.46)	< 0.001	1.26 (1.15‒1.38)	< 0.001	1.06 (0.90‒1.25)	0.49		
Previous coronary artery bypass graft	2.17 (1.29‒3.67)	0.004	1.71 (1.01‒2.89)	0.05	1.38 (0.34‒5.50)	0.65		
Previous stroke	2.13 (2.01‒2.26)	< 0.001	1.70 (1.60‒1.81)	< 0.001	0.84 (0.72‒0.98)	0.03	0.80 (0.68‒0.94)	0.007
Previous intracranial hemorrhage	1.64 (1.34‒2.00)	< 0.001			0.93 (0.57‒1.51)	0.76		
Heart failure	1.24 (1.17‒1.32)	< 0.001			1.17 (1.04‒1.31)	0.01		
Peripheral artery disease	1.65 (1.49‒1.83)	< 0.001	1.15 (1.04‒1.28)	0.01	0.95 (0.72‒1.25)	0.72		
Atrial fibrillation	1.41 (1.29‒1.53)	< 0.001			1.08 (0.90‒1.29)	0.44		
Chronic kidney disease	1.38 (1.30‒1.46)	< 0.001	1.13 (1.06‒1.20)	< 0.001	0.94 (0.82‒1.08)	0.37		
Chronic obstructive pulmonary disease	1.90 (1.72‒2.09)	< 0.001	1.31 (1.18‒1.45)	< 0.001	0.78 (0.59‒1.03)	0.08		
Malignancy	1.42 (1.28‒1.57)	< 0.001	1.12 (1.00‒1.24)	0.05	1.03 (0.83‒1.29)	0.76		
Chronic liver disease	1.32 (1.24‒1.40)	< 0.001			1.18 (1.04‒1.33)	0.01	1.22 (1.08‒1.39)	0.002
Peptic ulcer disease	1.35 (1.27‒1.43)	< 0.001			0.92 (0.80‒1.05)	0.22		
Dementia	1.81 (1.67‒1.97)	< 0.001	1.16 (1.06‒1.27)	0.001	0.75 (0.59‒0.95)	0.02	0.77 (0.61‒0.99)	0.04
Connective tissue disease	0.97 (0.83‒1.14)	0.73			1.02 (0.76‒1.35)	0.92		
Rhabdomyolysis	2.02 (1.32‒3.11)	0.001			1.52 (0.49‒4.70)	0.47		
Charlson Comorbidity Index	1.12 (1.11‒1.13)	< 0.001	1.03 (1.01‒1.04)	0.001	1.04 (1.01‒1.06)	0.004	1.09 (1.05‒1.12)	< 0.001
Number of implanted stents	1.08 (1.05‒1.10)	< 0.001	1.08 (1.05‒1.10)	< 0.001	1.01 (0.97‒1.06)	0.69		
Clinical diagnosis: acute myocardial infarction (vs. angina pectoris)	0.95 (0.91‒0.99)	0.02	1.14 (1.08‒1.19)	< 0.001	0.95 (0.88‒1.02)	0.17		
Hospital: primary or secondary center (vs. tertiary)	1.13 (1.09‒1.18)	< 0.001	0.90 (0.86‒0.93)	< 0.001	1.02 (0.95‒1.10)	0.56		
Year of the index percutaneous coronary intervention (vs. 2012)								
2013	0.97 (0.90‒1.05)	0.49			0.92 (0.80‒1.06)	0.24		
2014	0.97 (0.90‒1.04)	0.40			0.83 (0.73‒0.95)	0.006	0.76 (0.66‒0.87)	< 0.001
2015	0.96 (0.89‒1.03)	0.28			0.82 (0.72‒0.93)	0.003	0.73 (0.64‒0.84)	< 0.001
2016	1.00 (0.92‒1.08)	0.95			0.78 (0.68‒0.89)	< 0.001	0.70 (0.61‒0.81)	< 0.001
2017	0.98 (0.90‒1.07)	0.61			0.61 (0.52‒0.72)	< 0.001	0.55 (0.46‒0.65)	< 0.001
Good adherence (vs. poor)	0.51 (0.48‒0.54)	< 0.001	0.56 (0.52‒0.59)	< 0.001	1.49 (1.25‒1.78)	< 0.001	1.49 (1.25‒1.77)	< 0.001

^*^Composite of all-cause death, any revascularization, or stroke

### Subgroup analysis

**Fig. 3:**
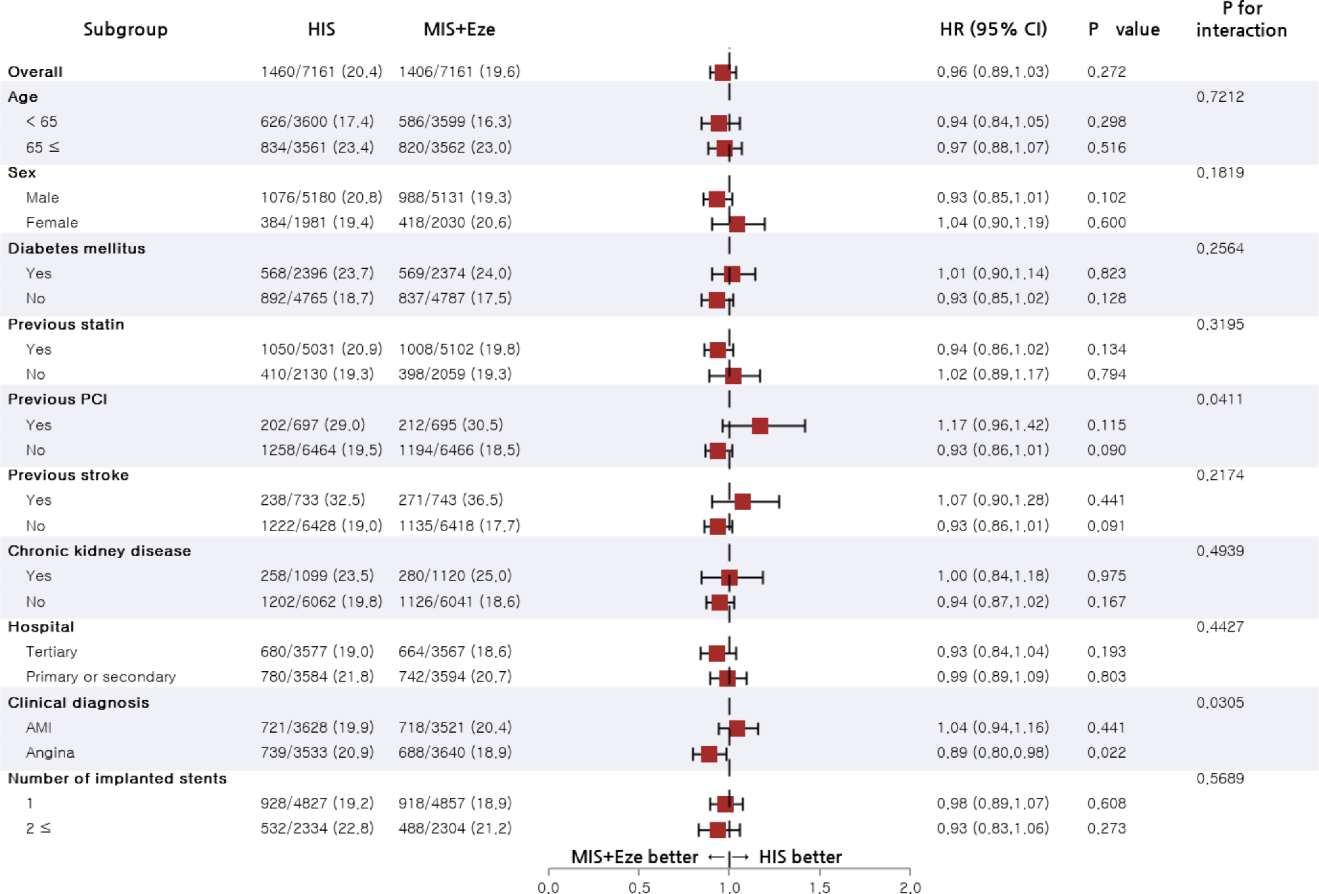
Subgroup analysis for risk of major adverse cardiac cerebrovascular events in the propensity score-matched population. Abbreviation: AMI, acute myocardial infarction; HIS, high-intensity statin; MIS + Eze, moderate-intensity statin plus ezetimibe; PCI, percutaneous coronary intervention

**Fig. 4:**
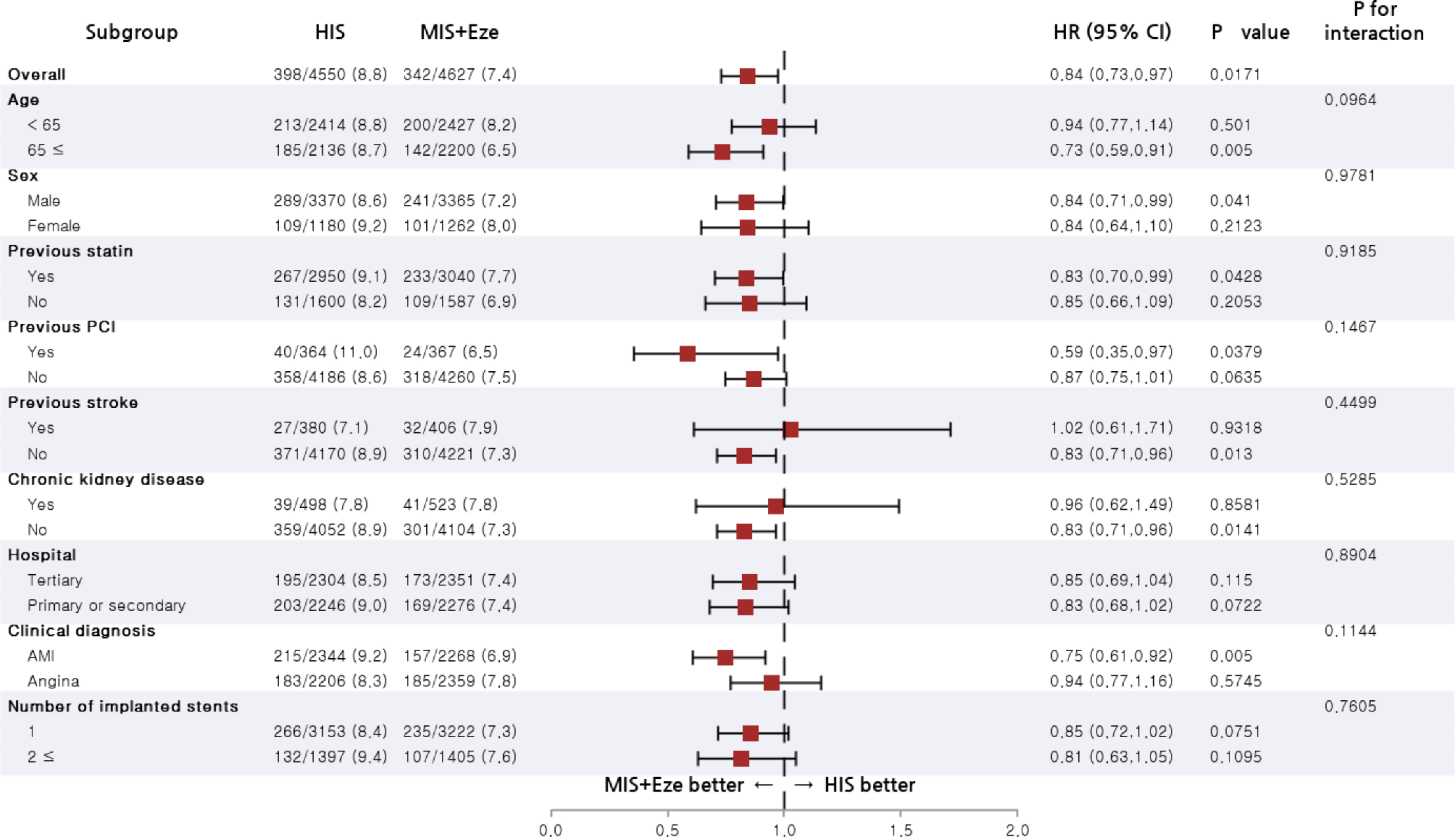
Subgroup analysis for a risk of new-onset diabetes mellitus in the propensity score-matched population. Abbreviation: AMI, acute myocardial infarction; HIS, high-intensity statin; MIS + Eze, moderate-intensity statin plus ezetimibe; PCI, percutaneous coronary intervention

For the subgroup analyses, patients were stratified by age, sex, and important comorbidities. Figures 3 and 4 present forest plots showing MACCE and new-onset DM related to various patients in propensity-score matched cohorts. The risk of MACCE was lower in the moderate-intensity statin plus ezetimibe group compared to the high-intensity statin group among patients who underwent PCI due to angina (HR, 0.89; 95% CI, 0.80–0.98). The impact of high-intensity statin therapy on the risk of new-onset DM was consistent across all subgroups.

### Statin adherence and clinical outcomes

The rate of poor drug adherence was higher in the high-intensity statin group (7.8%; 2,993/38,340) compared to the moderate-intensity statin plus ezetimibe group (4.9%; 352/7,161) (*P* < 0.001). Clinical outcomes according to drug adherence were listed in Supplement Table 4. Among patients with good adherence, the baseline characteristics (Supplementary Table 5) and clinical outcomes (Supplementary Table 6) comparing the moderate-intensity statin plus ezetimibe group with the high-intensity statin group were consistent with those observed in the overall population. (Supplementary Fig. 1) In the subgroup analyses of patients with good adherence, there were no significant differences between the groups in MACCE and new-onset DM (Supplementary Figs. 2 and 3).

## Discussion

In this large population-based study, we found that initiating a combination regimen of moderate-intensity statin and ezetimibe was associated with outcomes comparable to those observed with high-intensity statin monotherapy regarding long-term risk reduction of MACCE among patients undergoing PCI. Furthermore, our study demonstrated that long-term maintenance of moderate-intensity statin combined with ezetimibe was associated with significantly better drug adherence and a lower incidence of new-onset DM compared to high-intensity statin monotherapy.

High-intensity statin therapy, recommended for high-risk patients, involves using high doses of statins to achieve rapid and substantial reductions in LDL-C levels. However, half of the patients treated with high-intensity statin experience LDL-C reduction of less than 50% [11], and many patients may not reach the recently recommended LDL-C targets even after a 50% reduction [12]. Ezetimibe can provide additive benefits in lowering LDL-C and reducing the risk of cardiovascular events when combined with statins [13, 14]. This combination of statin and ezetimibe is currently recommended for patients who do not achieve the desired LDL-C targets with statins alone [1, 15]. 

High-intensity statin therapy and low to moderate-intensity statin therapy combined with ezetimibe are two different approaches that can be used to treat cardiovascular disease. Although high-intensity statins are essential for lowering LDL-C, they are associated with more side effects than lower-intensity statins [16]. Reducing the dose from a high-intensity statin to a moderate-intensity statin may modulate the side effects of a high-intensity statin, while adding ezetimibe to a moderate-intensity statin can maintain the LDL-C lowering capacity similar to a high-intensity statin. Adding ezetimibe to low or moderate-intensity statins showed similar or superior LDL-C lowering capacity compared to high-intensity statins [17, 18]. Though achieving an excellent reduction of LDL-C, a small study reported that platelet reactivity and proinflammatory chemokine were reduced more by the high-intensity than by ezetimibe plus lower-intensity statin [19]. A large, randomized controlled trial, RACING (randomized comparison of efficacy and safety of lipid-lowering with statin monotherapy versus statin/ezetimibe combination for high-risk cardiovascular diseases) trial, demonstrated the clinical effectiveness of moderate-intensity statin with ezetimibe combination therapy, which was non-inferior to high-intensity statin monotherapy in the 3-year risk of MACCE among high-risk patients [20]. Our study also revealed a consistent outcome whereby both the combination regimen of moderate-intensity statin and ezetimibe and high-intensity statin monotherapy was associated with a similar risk reduction of MACCEs among patients undergoing PCI, as demonstrated by a large population-based real-world data analysis. Risk reduction achieved through LDL-C lowering, rather than statin intensity alone, is now supported by multiple studies, including earlier trials and meta-analyses, as well as recent randomized controlled trials with non-statin therapies [21–24]. 

The safety of combination therapy of moderate-intensity statin and ezetimibe compared to high-intensity statin monotherapy is not well established. Increased serum statin concentrations or reduced body muscle mass increase the risk of statin-associated adverse events. Higher statin dose, advanced age, female sex, and lower body mass index are probable risk factors for statin-associated adverse events [25]. Adding ezetimibe to moderate-intensity statins (simvastatin and pitavastatin) did not increase the incidence of statin-associated adverse events or drug discontinuation [13, 14]. The combination of ezetimibe and lower-intensity statin caused lower incidences of drug-related adverse events (mainly muscle symptoms) [26] and discontinuation or dose reduction caused by intolerance [20] compared to high-intensity statin monotherapy. Because discontinuation of statin is associated with worse clinical outcomes [27], a combination of lower-intensity statin and ezetimibe can be preferred in patients at high risk of statin-associated adverse events. Our study highlights the importance of drug adherence in achieving favorable clinical outcomes. Poor adherence was more common in the high-intensity statin group than in the moderate-intensity statin plus ezetimibe group. Among patients with good adherence to their prescribed regimens, there was a significantly lower risk of MACCE and new-onset diabetes, emphasizing the critical role of adherence in optimizing cardiovascular outcomes. However, while the rate of poor adherence was approximately 3% higher in the high-intensity statin group, this difference did not significantly impact clinical outcomes, possibly due to the low overall proportion of poor adherence and other unmeasured factors that may have influenced results. These findings underscore the need for strategies to improve adherence to prescribed lipid-lowering therapies.

Although not causing acute symptoms, impaired glucose homeostasis by statins is one of the critical side effects. In a meta-analysis of randomized control trials, high-intensity statin therapy was associated with an increased risk of new-onset DM compared with moderate-intensity statin therapy [3]. Meanwhile, adding ezetimibe did not affect glucose metabolism [28], and no harmful effect was observed at very low LDL-C attained with add-on treatment with ezetimibe [29]. In contrast, a large genetic study that assessed life-long exposure to lower LDL-C levels due to carriage of genetic variants of NPC1L1, the target of ezetimibe, showed an increased risk of diabetes and suggested that on glucose homeostasis may be a class effect of LDL-C lowering agents [30]. There has been scarce clinical data on the effect of new-onset DM by long-term ezetimibe combination therapy. Although an observational study, our large clinical data show the benefit of the ezetimibe combination and dose reduction of statin on the development of DM in patients who received PCI.

The IMProved Reduction of Outcomes: Vytorin Efficacy International (IMPROVE-IT) trial, published in 2015 [20], demonstrated significant cardiovascular benefits of adding ezetimibe to moderate-intensity statin therapy, likely influencing clinical practice in our study population. During the study period from 2012 to 2017, the use of moderate-intensity statin plus ezetimibe increased notably, from 10.7% in 2015 to 20.4% in 2016, and further to 30.4% in 2017. Despite this increased adoption of the combination therapy, the efficacy of moderate-intensity statin plus ezetimibe in preventing MACCE and its impact on new-onset diabetes rates remained consistent over time compared to high-intensity statin therapy. This stability suggests that while the IMPROVE-IT trial may have encouraged more frequent use, the combination therapy maintained comparable benefits and safety outcomes across the years studied.

Our study has limitations. First, we could not identify reasons for combination therapy or monotherapy in this study. Patients receiving high-intensity statin monotherapy may have less severe risk profiles to tolerate high-intensity stains compared with those with combination therapy. Since ezetimibe combination treatment showed similar effectiveness as monotherapy despite the patients’ worse profile, this bias is unlikely to explain our findings. Second, the study’s generalizability may be limited by the selective sample, as only 15% of PCI patients were included. From 2012 to 2017, low- to moderate-intensity statins were commonly prescribed post-PCI, unlike current practices. This led to a smaller cohort focused on patients using high-intensity statins or moderate-intensity statins plus ezetimibe, and only those with sustained adherence were selected to enable a precise comparison of efficacy and safety.　Third, there is a potential selection bias due to unrecorded confounders, such as body mass index, left ventricular systolic function, angiographic severity, anthropometric and behavioral factors, and limited information on disease management based on claims. Fourth, the lack of a placebo comparator limits the ability to assess absolute glycemic effects solely attributable to statin or ezetimibe use. Additionally, potential differences in glycemic outcomes across treatment strategies—such as varying statin intensities or the addition of ezetimibe—require further investigation to clarify their specific impact on glucose metabolism. Finally, there was no information in our study about baseline and on-treatment LDL-C levels. Due to the absence of baseline and on-treatment LDL-C data, our analysis does not address LDL-C changes directly, limiting our ability to evaluate the differential effects of the treatment strategies on LDL-C levels and their potential impact on MACCE and new-onset DM risks. However, the majority of patients with cardiovascular disease or at high risk of cardiovascular disease in Korea have LDL-C levels of 120 ~ 140 mg/dL without prior statin treatment [31, 32]. The difference in the LDL-C level achieved after treatment between the two treatment strategies would be similar to the previous randomized controlled trial conducted in Korea [17, 18, 20]. 

## Conclusions

In patients undergoing PCI, moderate-intensity statin plus ezetimibe treatment was associated with a similar risk of MACCEs but a lower risk of new-onset DM compared to high-intensity statin alone. This study suggests that upfront combination treatment of moderate-intensity statin and ezetimibe may provide a feasible lipid-lowering alternative to high-intensity statins after PCI.

## Electronic supplementary material

Below is the link to the electronic supplementary material.

Supplementary Material 1

Supplementary Material 2

Supplementary Material 3

Supplementary Material 4

## Data Availability

The data that support the findings of this study are available from Korea Health Insurance Review and Assessment but restrictions apply to the availability of these data, which were used under license for the current study, and so are not publicly available. Data are however available from the authors upon reasonable request and with permission of Korea Health Insurance Review and Assessment.

## Abbreviations

LDL-C: Low-density lipoprotein-cholesterol
PCI: Percutaneous coronary intervention
HIRA: Health Insurance Review and Assessment
ICD-10: International Classification of Diseases 10th Revision
MACCE: Major adverse cardiac cerebrovascular event
DM: Diabetes mellitus
HR: Hazard ratio
CI: Confidence interval
