# Supplementary material for: Efficacy and diabetes risk of moderate-intensity statin plus ezetimibe versus high-intensity statin after percutaneous coronary intervention
Source: Cardiovasc Diabetol. 2024 Nov 5;23:396. doi: 10.1186/s12933-024-02498-3 (PMC11536862; doi:10.1186/s12933-024-02498-3)
Supplement: Supplementary file 1 — Supplementary Material 1 [file 12933_2024_2498_MOESM1_ESM.docx]

**Supplementary Table 1.** Definitions of comorbidities and clinical outcomes

| Variables | ICD-10 or procedural code | Diagnostic definition |
| --- | --- | --- |
| **Comorbidities** |  |  |
| Hypertension with medications | I10‒13, and I15 | Admission and out-patient clinic before index PCI date and anti-hypertensive medications ≥30 days |
| Diabetes mellitus with medications | E10‒14 | Admission and out-patient clinic before index PCI date and anti-diabetic medications or insulin ≥30 days |
| Previous statin | E78 | Admission and out-patient clinic before index PCI date and statin and/or ezetimibe ≥30 days |
| Previous myocardial infarction | I21‒22, and I252 | Admission before index PCI date |
| Previous PCI | M6551‒6554, M6561‒6567, M6571, M6572, and M6638 | Admission before index PCI date |
| Previous coronary artery bypass graft | O1640, O1641, O1647‒1649, OA640, OA641, and OA647‒649 | Admission before index PCI date |
| Previous ischemic stroke | G458, G459, I63, and I64 | Admission before index PCI and brain imaging |
| Previous intracranial hemorrhage | I690, I691, and I692 | Admission before index PCI and brain imaging |
| Heart failure | I11, I13, I42, I50, and I971 | Admission and out-patient clinic before index PCI date |
| Atrial fibrillation | I48 | Admission and out-patient clinic before index PCI date |
| Peripheral artery disease | I70, and I73 | Admission before index PCI date |
| Chronic kidney disease | N02‒08, N11, N12, N15, N16, N18, and N19 | Admission and out-patient clinic before index PCI date |
| Chronic obstructive pulmonary disease | J43, and J44 | Admission and out-patient clinic before index PCI date |
| Malignancy | C1‒C9 (excluding C43 and C73) | Admission before index PCI date |
| Chronic liver disease | B18, K70, K71, K73, K74, and K76 | Admission and out-patient clinic before index PCI date |
| Peptic ulcer disease | K25‒K28 | Admission and out-patient clinic before index PCI date |
| Dementia | F00‒03, G30, and G311 | Admission and out-patient clinic before index PCI date |
| Connective tissue disease | M30‒36 | Admission and out-patient clinic before index PCI date |
| Rhabdomyolysis | N178 and M6298 | Admission before index PCI date |
| Clinical diagnosis at the index procedure |  |  |
| Acute myocardial infarction | I21 and I22 | ICD-10-CM code at the index admission |
| Angina pectoris | I20, I24, and I25 | ICD-10-CM code at the index admission |
| Number of implanted stents |  | Number of implanted stents within 30 days from the index PCI |
| **Clinical outcomes** |  |  |
| All-cause death |  | No medical claims for >365 days nationwide |
| PCI by myocardial infarction | I21, I22 & M6551‒6554, M6561‒6567, M6571, M6572, and M6638 | Admission 30 days after from the index PCI |
| PCI by angina | I20, I24, I25 & M6551‒6554, M6561‒6567, M6571, M6572, and M6638 | Admission 30 days after from the index PCI |
| Coronary artery bypass graft | O1640, O1641, O1647‒1649, OA640, OA641, and OA647‒649 | Admission 30 days after from the index PCI |
| Transient ischemic attack | G458 and G459 | Admission after the index PCI and brain imaging |
| Cerebral infarction | I63 and I64 | Admission after the index PCI and brain imaging |
| New-onset diabetes mellitus with medications | E10‒14 | Admission and out-patient clinic 30 days after from the index PCI and anti-diabetic medications or insulin ≥30 days |
| Intracranial hemorrhage | I60‒I62 | Admission after the index PCI and brain imaging |
| Hepatoprotective agents | B18, K70, K71, K73, K74, and K76 | Admission and out-patient clinic after the index PCI |
| Rhabdomyolysis | N178 and M6298 | Admission after the index PCI |

Abbreviation: ICD, international classification of diseases; PCI, percutaneous coronary intervention.

**Supplementary Table 2.** List of medications

| Medications | Name | ATC code |
| --- | --- | --- |
| High-intensity statin | Atorvastatin 40mg or 80mg, Rosuvastatin 20mg or 40mg, and combinations | A10BD, A10BH52, C10AA05, C10AA07, C10BX |
| Moderate-intensity statin | Simvastatin 20mg or 40mg, Lovastatin 40mg, Pravastatin 40 or 80mg, Fluvastatin 80mg, Atorvastatin 10mg or 20mg, Rosuvastatin 5mg or 10mg, Pitavastatin 1, 2, or 4mg, and combinations | A10BD, A10BH52, C10AA01, C10AA02, C10AA03, C10AA04, C10AA05, C10AA07, C10AA08, C10BA06, C10BA02, C10BA05, C10BA06, C10BX |
| Low-intensity statin | Simvastatin 5mg or 10mg, Lovastatin 20mg, Pravastatin 5, 10, or 20mg, Fluvastatin 20 or 40mg, and combinations | C10AA01, C10AA02, C10AA03, C10AA04, C10BX |
| Ezetimibe | Ezetimibe and combinations | C10AX10, C10BA02, C10BA05, C10BA06, C10BX |
| Other lipid lowering agents | Bezafibrate, Gemfibrozil, Fenofibrate, Nicotinic acid, Omega-3 fatty acids, Evolocumab, Alirocumab, and combinations | C10AB02, C10AB04, C10AB05, C10AB11, C10AD02, C10AX06, C10AX13, C10AX14, C10BA03, C10BA07, C10BA08, C10BA12 |
| Anti-diabetic medications or insulin | Insulins and analogues, Biguanides, Sulfonylureas, Alpha glucosidase inhibitors, Thiazolidinediones, Dipeptidyl peptidase 4 inhibitors, Glucagon-like peptide-1 analogues, Sodium-glucose co-transporter 2 inhibitors, and combinations | A10AB, A10AC, A10AD, A10AE, A10AF, A10BA, A10BB, A10BD, A10BF, A10BG, A10BH, A10BJ, A10BK |
| Aspirin | Aspirin | B01AC06, B01AC30, N02BA01, N02BA51 |
| Clopidogrel | Clopidogrel | B01AC04, B01AC30 |
| Prasugrel | Prasugrel | B01AC22 |
| Ticagrelor | Ticagrelor | B01AC24 |
| Beta-blocker | Propranolol, Nadolol, Carteolol, Tertatolol, Cloranolol, Metoprolol, Atenolol, Acebutolol, Betaxolol, Bevantolol, Bisoprolol, Celiprolol, Esmolol, s-Atenolol, Nebivolol, Labetalol, Carvedilol, and combinations | C07AA05, C07AA12, C07AA15, C07AA16, C07AA27, C07AB02, C07AB03, C07AB04, C07AB05, C07AB06, C07AB07, C07AB08, C07AB09, C07AB11, C07AB12, C07AG, C07AG01, C07AG02, C07BB02, C07BB04, C07BB07, C07CB03, C07FB02, C10BX |
| Renin-angiotensin system inhibitor | Captopril, Enalapril, Lisinopril, Perindopril, Ramipril, Cilazapril, Moexipril, Zofenopril, Imidapril, Losartan, Eprosartan, Valsartan, Irbesartan, Candesartan, Telmisartan, Olmesartan, Azilsartan, Fimasartan, and combinations | C09AA01, C09AA02, C09AA03, C09AA04, C09AA05, C09AA08, C09AA13, C09AA15, C09AA16, C09CA01, C09CA02, C09CA03, C09CA04, C09CA06, C09CA07, C09CA08, C09CA09, C09CA10, C09DA, C09DB, C09DX, C10BX |
| Warfarin | Warfarin | B01AA03 |
| Direct oral anticoagulants | Dabigatran, Rivaroxaban, Apixaban, and Edoxaban | B01AE07, B01AF01, B01AF02, B01AF03 |
| Other anti-hypertensive medications | Methyldopa, Prazosin, Doxazosin, Hydralazine, Minoxidil, Nitroprusside, Hydrochlorothiazide, Chlortalidone, Indapamide, Furosemide, Torasemide, Spironolactone, Eplerenone, Finerenone, Amiloride, Ifenprodil, Amlodipine, Felodipine, Nicardipine, Nifedipine, Nimodipine, Lacidipine, Manidipine, Barnidipine, Lercanidipine, Cilnidipine, Benidipine, Verapamil, Diltiazem, and combinations | C02AB, C02CA01, C02CA04, C02DB02, C02DC01, C02DD01, C03AA03, C03AX01, C03BA04, C03BA11, C03CA01, C03CA04, C03DA01, C03DA04, C03DA05, C03DB01, C04AX28, C08CA01, C08CA02, C08CA04, C08CA05, C08CA06, C08CA09, C08CA11, C08CA12, C08CA13, C08CA14, C08CA15, C08DA01, C08DB01, C09DA, C09DB, C09DX |
| Hepatoprotective agents | Ursodeoxycholic acid, Biphenyl dimethyl dicarboxylate, Silymarin, and L-Ornithine-L-Aspartate | A05AA02, A05BA, A05BA03, A05BA06 |

Abbreviation: ATC, anatomical therapeutic chemical.

**Supplementary Table 3.** Clinical outcomes in the crude population

| Endpoints | Cumulative incidence^*^ | |  | Incidence rate per 100 person-year | | Log-Rank *P* value | Unadjusted HR (95% CI) | *P* value | Adjusted HR (95% CI) | *P* value |
| --- | --- | --- | --- | --- | --- | --- | --- | --- | --- | --- |
|  | High-intensity statin  (N = 38,340) | Moderate-intensity statin plus ezetimibe  (N = 7,161) |  | High-intensity statin  (N = 38,340) | Moderate-intensity statin plus ezetimibe  (N = 7,161) |  |  |  |  |  |
| **Efficacy endpoints** |  |  |  |  |  |  |  |  |  |  |
| Major adverse cardiac cerebrovascular events^†^ | 7,611 (32.5) | 1,406 (31.9) |  | 6.9 | 6.8 | 0.63 | 0.99 (0.93‒1.04) | 0.63 | 0.95 (0.89‒1.01) | 0.08 |
| All-cause death | 2,437 (14.7) | 448 (13.9) |  | 2.0 | 2.0 | 0.44 | 0.96 (0.87‒1.06) | 0.44 | 0.95 (0.85‒1.05) | 0.32 |
| Revascularization | 4,460 (17.3) | 781 (16.8) |  | 4.0 | 3.7 | 0.07 | 0.93 (0.86‒1.01) | 0.07 | 0.92 (0.85‒0.99) | 0.03 |
| Percutaneous coronary intervention by myocardial infarction | 1,003 (4.2) | 115 (2.9) |  | 0.8 | 0.5 | <0.001 | 0.61 (0.50‒0.74) | <.0001 | 0.80 (0.65‒0.97) | 0.03 |
| Percutaneous coronary intervention by angina | 3,412 (12.9) | 659 (13.8) |  | 3.0 | 3.1 | 0.38 | 1.04 (0.96‒1.13) | 0.38 | 0.96 (0.88‒1.04) | 0.30 |
| Coronary artery bypass graft | 80 (0.4) | 12 (0.4) |  | 0.1 | 0.1 | 0.47 | 0.80 (0.44‒1.47) | 0.47 | 0.68 (0.36‒1.28) | 0.23 |
| Ischemic stroke | 1,705 (7.5) | 396 (8.9) |  | 1.4 | 1.8 | <0.001 | 1.25 (1.12‒1.40) | <.0001 | 1.02 (0.91‒1.14) | 0.72 |
| Transient ischemic attack | 322 (1.5) | 80 (2.0) |  | 0.3 | 0.4 | 0.02 | 1.33 (1.04‒1.70) | 0.02 | 1.15 (0.89‒1.48) | 0.30 |
| Cerebral infarction | 1,451 (6.3) | 339 (7.5) |  | 1.2 | 1.5 | <0.001 | 1.26 (1.12‒1.42) | <.0001 | 1.01 (0.90‒1.15) | 0.83 |
| **Safety endpoints** |  |  |  |  |  |  |  |  |  |  |
| New-onset diabetes mellitus with medications | 2,371 (13.2) | 342 (10.7) |  | 3.0 | 2.5 | 0.001 | 0.83 (0.74‒0.93) | 0.001 | 0.83 (0.73‒0.93) | 0.002 |
| Intracranial hemorrhage | 602 (2.3) | 97 (2.3) |  | 0.4 | 0.4 | 0.71 | 1.04 (0.85‒1.28) | 0.71 | 1.09 (0.88‒1.23) | 0.43 |
| Hepatoprotective agents | 2,363 (9.2) | 396 (10.2) |  | 1.6 | 1.8 | 0.08 | 1.10 (0.99‒1.21) | 0.07 | 1.11 (1.00‒1.23) | 0.05 |
| Rhabdomyolysis | 178 (0.9) | 27 (0.8) |  | 0.2 | 0.1 | 0.30 | 0.81 (0.54‒1.21) | 0.30 | 0.72 (0.47‒1.09) | 0.12 |

^*^Events reported as number (6-year Kaplan-Meier estimate)

^†^Composite of all-cause death, revascularization, or stroke

**Supplementary Table 4.** Clinical outcomes according to drug adherence

| Endpoints | Cumulative incidence^*^ | |  | Incidence rate per 100 person-year | | Log-Rank *P* value | Unadjusted HR (95% CI) | *P* value | Adjusted HR (95% CI) | *P* value |
| --- | --- | --- | --- | --- | --- | --- | --- | --- | --- | --- |
|  | Poor  Adherence  (N = 3,345) | Good  Adherence  (N = 42,156) |  | Poor  Adherence  (N = 3,345) | Good  Adherence  (N = 42,156) |  |  |  |  |  |
| **Efficacy endpoints** |  |  |  |  |  |  |  |  |  |  |
| Major adverse cardiac cerebrovascular events^†^ | 1,185 (58.3) | 7,832 (30.0) |  | 12.8 | 6.5 | <0.001 | 0.51 (0.48‒0.54) | <0.001 | 0.56 (0.52‒0.59) | <0.001 |
| All-cause death | 725 (40.2) | 2,160 (12.2) |  | 6.9 | 1.6 | <0.001 | 0.23 (0.21‒0.25) | <0.001 | 0.29 (0.26‒0.31) | <0.001 |
| Revascularization | 447 (25.6) | 4,794 (16.6) |  | 4.6 | 3.8 | <0.001 | 0.84 (0.76‒0.93) | <0.001 | 0.85 (0.77‒0.94) | 0.001 |
| Percutaneous coronary intervention by myocardial infarction | 158 (9.6) | 960 (3.6) |  | 1.6 | 0.7 | <0.001 | 0.47 (0.40‒0.56) | <0.001 | 0.52 (0.44‒0.62) | <0.001 |
| Percutaneous coronary intervention by angina | 282 (15.5) | 3,789 (12.9) |  | 2.8 | 3.0 | 0.30 | 1.07 (0.95‒1.20) | 0.30 | 1.05 (0.93‒1.19) | 0.40 |
| Coronary artery bypass graft | 22 (0.8) | 81 (0.4) |  | 0.1 | 0.1 | 0.08 | 0.57 (0.30‒1.07) | 0.08 | 0.56 (0.29‒1.05) | 0.07 |
| Ischemic stroke | 242 (13.7) | 1,859 (7.2) |  | 2.4 | 1.4 | <0.001 | 0.59 (0.52‒0.68) | <0.001 | 0.73 (0.63‒0.83) | <0.001 |
| Transient ischemic attack | 35 (2.3) | 367 (1.5) |  | 0.3 | 0.3 | 0.25 | 0.82 (0.58‒1.15) | 0.25 | 0.94 (0.66‒1.34) | 0.73 |
| Cerebral infarction | 216 (12.2) | 1,574 (6.0) |  | 2.1 | 1.2 | <0.001 | 0.56 (0.49‒0.65) | <0.001 | 0.70 (0.60‒0.81) | <0.001 |
| **Safety endpoints** |  |  |  |  |  |  |  |  |  |  |
| New-onset diabetes mellitus with medications | 131 (9.6) | 2,582 (13.0) |  | 2.0 | 3.0 | <0.001 | 1.49 (1.25‒1.78) | <0.001 | 1.49 (1.25‒1.77) | <0.001 |
| Intracranial hemorrhage | 81 (3.7) | 618 (2.2) |  | 0.7 | 0.4 | <0.001 | 0.56 (0.49‒0.64) | <0.001 | 0.66 (0.58‒0.75) | <0.001 |
| Hepatoprotective agents | 241 (11.1) | 2,518 (9.1) |  | 2.1 | 1.6 | <0.001 | 0.75 (0.70‒0.81) | <0.001 | 0.77 (0.72‒0.83) | <0.001 |
| Rhabdomyolysis | 33 (1.5) | 172 (0.9) |  | 0.3 | 0.1 | <0.001 | 0.40 (0.28‒0.58) | <0.001 | 0.52 (0.35‒0.76) | <0.001 |

^*^Events reported as number (6-year Kaplan-Meier estimate)

^†^Composite of all-cause death, revascularization, or stroke

**Supplementary Table 5.** Baseline characteristics in patients with good adherence

| Variables | Crude population | | |  | Propensity score-matched population | | | |
| --- | --- | --- | --- | --- | --- | --- | --- | --- |
|  | High-intensity statin (n = 35,347) | Moderate-intensity statin plus ezetimibe (n = 6,809) | *P* value |  | High-intensity statin (n = 6,809) | Moderate-intensity statin plus ezetimibe (n = 6,809) | *P* value | Standardized Difference |
|  |  |  |  |  |  |  |  |  |
| Age | 61.75 ± 11.26 | 63.95 ± 10.50 | <0.001 |  | 63.94 ± 10.43 | 63.95 ± 10.50 | 0.95 | 0.001 |
| Age |  |  | <0.001 |  |  |  | 0.94 |  |
| <55 | 9553 (27) | 1289 (18.9) |  |  | 1288 (18.9) | 1289 (18.9) |  |  |
| 55-64 | 11324 (32) | 2176 (32) |  |  | 2192 (32.2) | 2176 (32) |  |  |
| 65-74 | 9381 (26.5) | 2209 (32.4) |  |  | 2221 (32.6) | 2209 (32.4) |  |  |
| 75≤ | 5089 (14.4) | 1135 (16.7) |  |  | 1108 (16.3) | 1135 (16.7) |  |  |
| Male | 27233 (77) | 4882 (71.7) | <0.001 |  | 4859 (71.4) | 4882 (71.7) | 0.66 | 0.007 |
| Hypertension with medication | 22922 (64.8) | 5194 (76.3) | <0.001 |  | 5144 (75.5) | 5194 (76.3) | 0.32 | 0.017 |
| Diabetes mellitus with medication | 9569 (27.1) | 2242 (32.9) | <0.001 |  | 2307 (33.9) | 2242 (32.9) | 0.24 | -0.020 |
| Previous statin | 19476 (55.1) | 4888 (71.8) | <0.001 |  | 4874 (71.6) | 4888 (71.8) | 0.79 | 0.005 |
| Previous myocardial infarction | 2027 (5.7) | 572 (8.4) | <0.001 |  | 571 (8.4) | 572 (8.4) | 0.98 | 0.001 |
| Previous percutaneous coronary intervention | 1830 (5.2) | 664 (9.8) | <0.001 |  | 679 (10) | 664 (9.8) | 0.67 | -0.007 |
| Previous coronary artery bypass graft | 24 (0.1) | 10 (0.1) | 0.04 |  | 11 (0.2) | 10 (0.1) | 0.83 | -0.004 |
| Previous ischemic stroke | 3024 (8.6) | 690 (10.1) | <0.001 |  | 682 (10) | 690 (10.1) | 0.82 | 0.004 |
| Previous intracranial hemorrhage | 238 (0.7) | 63 (0.9) | 0.02 |  | 63 (0.9) | 63 (0.9) | 1.00 | 0.000 |
| Heart failure | 3898 (11) | 954 (14) | <0.001 |  | 963 (14.1) | 954 (14) | 0.82 | -0.004 |
| Atrial fibrillation | 912 (2.6) | 297 (4.4) | <0.001 |  | 280 (4.1) | 297 (4.4) | 0.47 | 0.012 |
| Peripheral artery disease | 1564 (4.4) | 433 (6.4) | <0.001 |  | 428 (6.3) | 433 (6.4) | 0.86 | 0.003 |
| Chronic kidney disease | 4018 (11.4) | 1041 (15.3) | <0.001 |  | 1058 (15.5) | 1041 (15.3) | 0.69 | -0.007 |
| Chronic obstructive pulmonary disease | 940 (2.7) | 182 (2.7) | 0.95 |  | 174 (2.6) | 182 (2.7) | 0.67 | 0.007 |
| Malignancy | 1197 (3.4) | 253 (3.7) | 0.17 |  | 246 (3.6) | 253 (3.7) | 0.75 | 0.005 |
| Chronic liver disease | 3688 (10.4) | 885 (13) | <0.001 |  | 870 (12.8) | 885 (13) | 0.70 | 0.007 |
| Peptic ulcer disease | 3452 (9.8) | 891 (13.1) | <0.001 |  | 887 (13) | 891 (13.1) | 0.92 | 0.002 |
| Dementia | 1293 (3.7) | 386 (5.7) | <0.001 |  | 399 (5.9) | 386 (5.7) | 0.63 | -0.008 |
| Connective tissue disease | 619 (1.8) | 141 (2.1) | 0.07 |  | 126 (1.9) | 141 (2.1) | 0.35 | 0.016 |
| Rhabdomyolysis | 41 (0.1) | 14 (0.2) | 0.06 |  | 14 (0.2) | 14 (0.2) | 1.00 | 0.000 |
| Charlson Comorbidity Index | 2.00 ± 1.75 | 2.41 ± 1.88 | <0.001 |  | 2.41 ± 1.88 | 2.41 ± 1.88 | 0.86 | 0.003 |
| Charlson Comorbidity Index |  |  | <0.001 |  |  |  | 0.34 |  |
| 0 | 7220 (20.4) | 959 (14.1) |  |  | 988 (14.5) | 959 (14.1) |  |  |
| 1 | 9022 (25.5) | 1537 (22.6) |  |  | 1470 (21.6) | 1537 (22.6) |  |  |
| 2 | 7664 (21.7) | 1489 (21.9) |  |  | 1553 (22.8) | 1489 (21.9) |  |  |
| 3≤ | 11441 (32.4) | 2824 (41.5) |  |  | 2798 (41.1) | 2824 (41.5) |  |  |
| Clinical diagnosis at the index procedure |  |  | <0.001 |  |  |  |  | 0.009 |
| Acute myocardial infarction | 17553 (49.7) | 1503 (22.1) |  |  | 1477 (21.7) | 1503 (22.1) | 0.59 |  |
| Angina pectoris | 17794 (50.3) | 5306 (77.9) |  |  | 5332 (78.3) | 5306 (77.9) |  |  |
| Number of implanted stents | 1.44 ± 0.87 | 1.38 ± 0.91 | <0.001 |  | 1.38 ± 0.87 | 1.38 ± 0.91 | 0.82 | 0.004 |
| Number of implanted stents |  |  | 0.02 |  |  |  | 0.74 |  |
| 1 | 23470 (66.4) | 4618 (67.8) |  |  | 4636 (68.1) | 4618 (67.8) |  |  |
| 2≤ | 11877 (33.6) | 2191 (32.2) |  |  | 2173 (31.9) | 2191 (32.2) |  |  |
| Hospital |  |  | 0.68 |  |  |  | 0.78 | 0.005 |
| Tertiary center | 17674 (50) | 3423 (50.3) |  |  | 3407 (50) | 3423 (50.3) |  |  |
| Primary or secondary center | 17673 (50) | 3386 (49.7) |  |  | 3402 (50) | 3386 (49.7) |  |  |
| Year of the index percutaneous coronary intervention |  |  | <0.001 |  |  |  | 0.80 |  |
| 2012 | 2740 (7.8) | 1031 (15.1) |  |  | 1082 (15.9) | 1031 (15.1) |  |  |
| 2013 | 3495 (9.9) | 902 (13.2) |  |  | 882 (13) | 902 (13.2) |  |  |
| 2014 | 6022 (17) | 689 (10.1) |  |  | 672 (9.9) | 689 (10.1) |  |  |
| 2015 | 7566 (21.4) | 719 (10.6) |  |  | 710 (10.4) | 719 (10.6) |  |  |
| 2016 | 8340 (23.6) | 1391 (20.4) |  |  | 1421 (20.9) | 1391 (20.4) |  |  |
| 2017 | 7184 (20.3) | 2077 (30.5) |  |  | 2042 (30) | 2077 (30.5) |  |  |
| Medication |  |  |  |  |  |  |  |  |
| Aspirin | 34916 (98.8) | 6518 (95.7) | <0.001 |  | 6546 (96.1) | 6518 (95.7) | 0.22 | -0.021 |
| Clopidogrel | 22809 (64.5) | 5372 (78.9) | <0.001 |  | 5397 (79.3) | 5372 (78.9) | 0.60 | 0.019 |
| Prasugrel | 2151 (6.1) | 424 (6.2) | 0.65 |  | 408 (6) | 424 (6.2) | 0.57 | 0.029 |
| Ticagrelor | 9956 (28.2) | 722 (10.6) | <0.001 |  | 741 (10.9) | 722 (10.6) | 0.60 | -0.057 |
| Beta-blocker | 27356 (77.4) | 4137 (60.8) | <0.001 |  | 4172 (61.3) | 4137 (60.8) | 0.54 | -0.011 |
| Renin-angiotensin system inhibitor | 24143 (68.3) | 3150 (46.3) | <0.001 |  | 3137 (46.1) | 3150 (46.3) | 0.82 | 0.004 |
| Warfarin | 806 (2.3) | 117 (1.7) | 0.004 |  | 124 (1.8) | 117 (1.7) | 0.65 | -0.008 |
| Direct oral anticoagulants | 544 (1.5) | 110 (1.6) | 0.64 |  | 108 (1.6) | 110 (1.6) | 0.89 | 0.002 |

Data is presented as mean ± standard deviation or number (percentage)

**Supplementary Table 6.** Clinical outcomes in patients with good adherence after the propensity score matching

| Endpoints | Cumulative incidence^*^ | |  | Incidence rate per 100 person-year | | Log-Rank *P* value | HR (95% CI) | *P* value |
| --- | --- | --- | --- | --- | --- | --- | --- | --- |
|  | High-intensity statin  (N = 6,809) | Moderate-intensity statin plus ezetimibe  (N = 6,809) |  | High-intensity statin  (N = 6,809) | Moderate-intensity statin plus ezetimibe  (N = 6,809) |  |  |  |
| **Efficacy endpoints** |  |  |  |  |  |  |  |  |
| Major adverse cardiac cerebrovascular events^†^ | 1,288 (31.8) | 1,293 (30.5) |  | 6.8 | 6.6 | 0.99 | 1.00 (0.93‒1.08) | 1.00 |
| All-cause death | 364 (13.3) | 376 (12.4) |  | 1.7 | 1.7 | 0.58 | 1.04 (0.90‒1.20) | 0.58 |
| Revascularization | 762 (17.5) | 738 (16.6) |  | 3.9 | 3.6 | 0.50 | 0.97 (0.87‒1.07) | 0.50 |
| Percutaneous coronary intervention by myocardial infarction | 100 (2.7) | 105 (2.7) |  | 0.5 | 0.5 | 0.70 | 1.05 (0.80‒1.39) | 0.70 |
| Percutaneous coronary intervention by angina | 652 (14.7) | 628 (13.8) |  | 3.3 | 3.1 | 0.48 | 0.96 (0.86‒1.07) | 0.48 |
| Coronary artery bypass graft | 16 (0.4) | 10 (0.3) |  | 0.1 | 0.1 | 0.24 | 0.63 (0.29‒1.38) | 0.25 |
| Ischemic stroke | 343 (9.5) | 365 (8.4) |  | 1.8 | 1.7 | 0.38 | 1.07 (0.92‒1.24) | 0.38 |
| Transient ischemic attack | 70 (1.9) | 78 (1.9) |  | 0.3 | 0.4 | 0.48 | 1.12 (0.81‒1.55) | 0.48 |
| Cerebral infarction | 292 (7.9) | 309 (7.0) |  | 1.5 | 1.5 | 0.47 | 1.06 (0.90‒1.25) | 0.47 |
| **Safety endpoints** |  |  |  |  |  |  |  |  |
| New-onset diabetes mellitus with medications | 389 (13.2) | 333 (11.0) |  | 3.2 | 2.5 | 0.01 | 0.83 (0.72‒0.97) | 0.02 |
| Intracranial hemorrhage | 83 (2.1) | 90 (2.2) |  | 0.4 | 0.4 | 0.54 | 1.08 (0.80‒1.46) | 0.60 |
| Hepatoprotective agents | 325 (9.4) | 366 (9.9) |  | 1.5 | 1.7 | 0.13 | 1.13 (0.98‒1.32) | 0.10 |
| Rhabdomyolysis | 29 (0.9) | 23 (0.8) |  | 0.2 | 0.1 | 0.41 | 0.80 (0.46‒1.38) | 0.41 |

^*^Events reported as number (6-year Kaplan-Meier estimate)

^†^Composite of all-cause death, revascularization, or stroke
