# Supplementary figures and images for: Efficacy and diabetes risk of moderate-intensity statin plus ezetimibe versus high-intensity statin after percutaneous coronary intervention
Source: Cardiovasc Diabetol. 2024 Nov 5;23:396. doi: 10.1186/s12933-024-02498-3 (PMC11536862; doi:10.1186/s12933-024-02498-3)

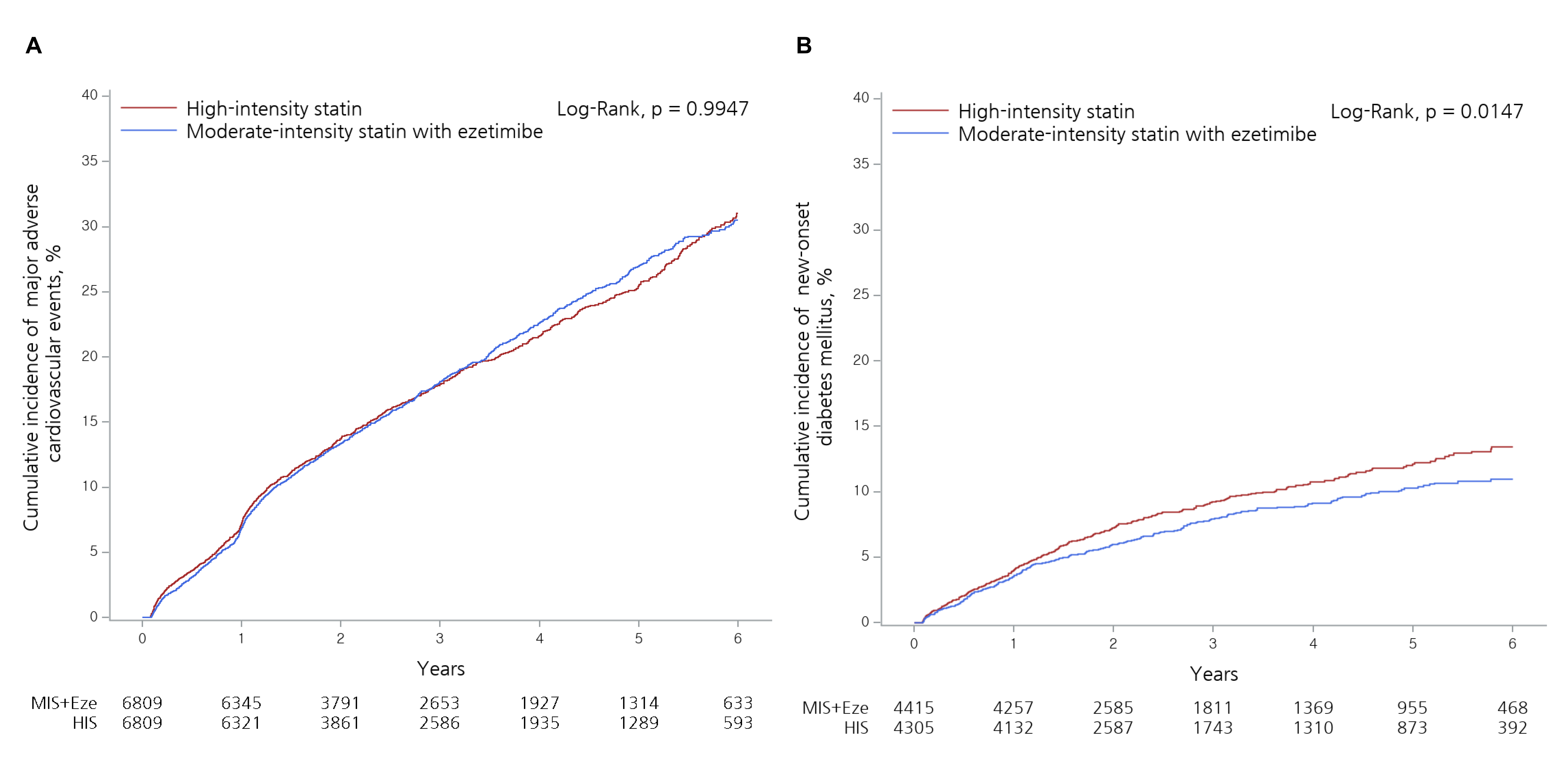

Supplement: Supplementary file 2 — Supplementary Material 2 [file 12933_2024_2498_MOESM2_ESM.tif]

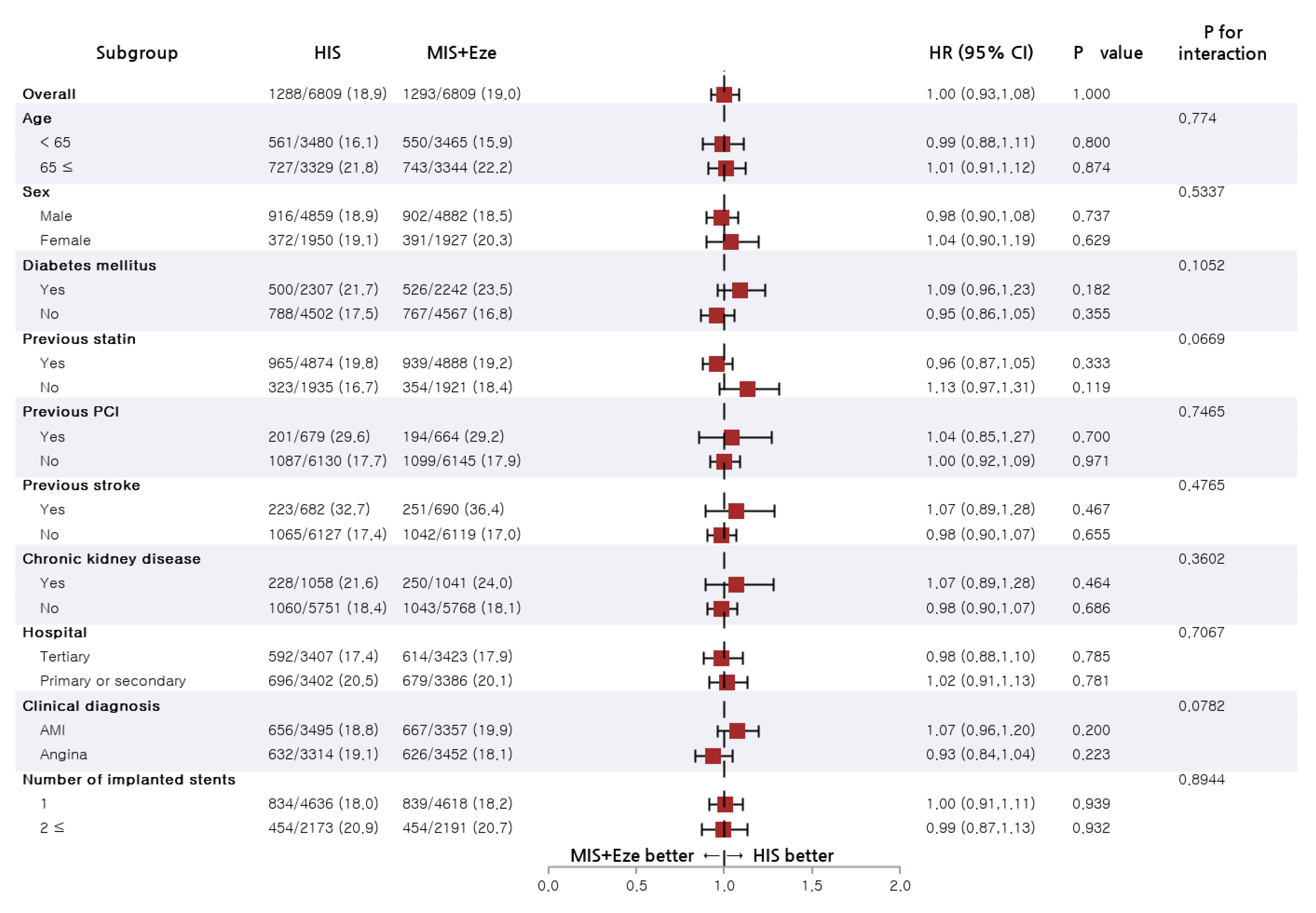

Supplement: Supplementary file 3 — Supplementary Material 3 [file 12933_2024_2498_MOESM3_ESM.tif]

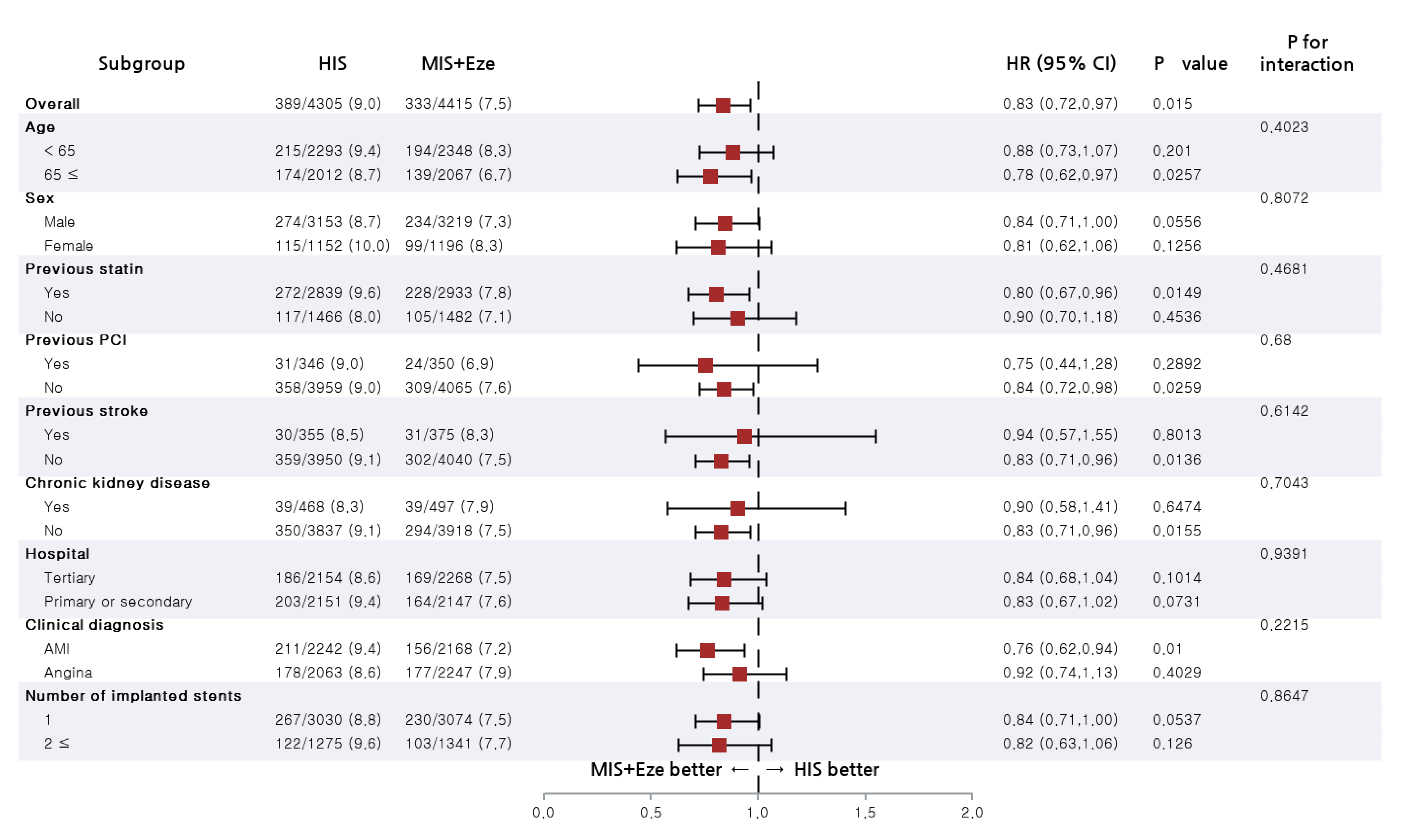

Supplement: Supplementary file 4 — Supplementary Material 4 [file 12933_2024_2498_MOESM4_ESM.tif]
